# Sheep fatigue during transport: Lost in translation?

**DOI:** 10.1017/awf.2024.13

**Published:** 2024-03-11

**Authors:** Katia Colitti, Malcolm Mitchell, Fritha Langford

**Affiliations:** 1 The University of Edinburgh, Royal Dick School of Veterinary Studies, Roslin, Midlothian, UK; 2 Scotland’s Rural College, Edinburgh, UK; 3 Newcastle University, School of Natural and Environmental Science, Newcastle-upon-Tyne, UK

**Keywords:** Animal welfare, anthropomorphism, live transport, qualitative research, reflexive thematic analysis, ruminant

## Abstract

Although sheep are commonly transported long distances, and sheep welfare during transport is a topic of research and policy discussion, the subject of their fatigue during transport has been under-researched. The current qualitative study, focused on the EU and UK, aimed to critically analyse stakeholder views on issues relating to sheep fatigue, including behavioural indications of fatigue, the interplay between fatigue and other factors, and the practicalities of identifying fatigue in commercial transport conditions. Insight into stakeholder perceptions of these issues could contribute to the body of knowledge regarding sheep fatigue during transport, potentially playing a part in future efforts to improve fatigue understanding and detection. Eighteen experts from different stakeholder groups were interviewed. Reflexive thematic analysis of interview data yielded four themes and three sub-themes. The first theme, “Let’s anthropomorphise it a little bit”, underscores the pervasiveness of anthropomorphism and suggests using it in a conscious and deliberate way to drive stakeholder engagement and policy change. The second theme, “We think that they’re like we are and they’re not”, cautions against wholesale transfer of human experiences to animals. The third theme, ‘See the whole animal’, advocates using Qualitative Behaviour Analysis (QBA), proven reliable in other contexts, to deepen and enrich our current understanding of fatigue. The fourth theme, ‘Fatigue “never comes up”’, highlights the fact that fatigue is rarely if ever discussed in the context of sheep transport. These themes suggest several avenues for future research, including developing QBA-based assessments for fatigue to improve welfare during transport.

## Introduction

Every year, large numbers of sheep are transported within the European Union (EU) and exported from the EU (European Parliamentary Research Service 2020). Within the EU, sheep are transported largely by road (European Court of Auditors [ECA] 2023). Transport from the EU (e.g. to the Middle East) occurs by sea (DG Health & Food Safety 2020). Sheep are transported for slaughter, fattening and breeding and, although no breakdown by transport purpose is available, it is believed that most sheep transported within the EU and exported are transported for immediate slaughter (Eurogroup for Animals 2019). Transport data available from various EU databases are fragmented and incomplete and real journey times are potentially much longer than shown in official data (Eurogroup for Animals & Compassion in World Farming [CWF] 2023). Of sheep and goats transported between EU countries over the 2017–2021 reference period, 55% experienced short journeys (currently defined as lasting up to 8 h), 42% experienced long journeys (over 8 h but fewer than 24 h) and 3% experienced very long journeys (over 24 h) (ECA 2023).

The distances travelled can be significant, particularly for exports from the EU. The most important intra-EU sheep transport routes are Romania-Greece, Romania-Italy, France-Italy, Hungary-Italy, and Spain-France; the most significant export routes are Romania and Spain to Libya, Jordan, Israel, and Lebanon (Eurogroup for Animals 2019). Transport routes are becoming longer due to factors such as differences in rearing and consumption regions and slaughterhouse consolidation (Eurogroup for Animals 2019).

Certain sources estimate that the majority of the approximately 85 million sheep kept in the EU (Eurostat 2019) will experience transport (Messori *et al.* 2015a). Since 2019, on average, around 3.5 million sheep per year were traded alive between EU countries (Nielsen *et al.* 2022). The estimates set out in Nielsen *et al.* (2022) exclude exports and imports from/into the EU which, taken together, are estimated to amount to an additional three million animals a year (ECA 2023) and are expected to continue to grow (Eurogroup for Animals 2019). Between October 2021 and April 2023, the EU exported over four million sheep to the Middle East and North Africa (Eurogroup for Animals & CWF 2023). The estimates also exclude intra-country transport. For example, in Great Britain, 12 million sheep a year are transported from farms for slaughter each year (Agriculture and Horticulture Development Board [AHDB] 2023). (This example is relevant as the UK was included in some of the datasets cited above.)

Despite significant and growing long-distance transport of millions of sheep each year, sheep fatigue does not receive much attention and remains poorly understood (Cockram *et al.* 2012). Fatigue can be defined as “[w]eariness or exhaustion from … exertion or stress” (Merriam-Webster), “difficulty in initiating or sustaining voluntary activities” (Tanaka & Watanabe 2012; p 727), or “physiological state representing extreme tiredness and exhaustion” (Nielsen *et al* 2022; p 18). Although exhaustion has been used as a synonym of fatigue (as in the definition above), in the context of this project and during interviews (detailed below), exhaustion has been understood to be the advanced stage of fatigue, equivalent to severe fatigue. Fatigue can be physical, e.g. pertaining to peripheral or central muscle activation (Tanaka & Watanabe 2012) or mental, experienced as increasing “weariness” caused by extended periods of cognitive exertion (Borghini *et al.* 2014; p 61; Russell *et al.* 2022).

The lack of focus on fatigue in sheep during transport may be at least partly explained by the common perception that sheep are hardy, resilient animals (Jones *et al.* 2010). There is ample evidence that transport *is* stressful for sheep (Cockram *et al.* 1996), even under conditions that follow best practices (Cockram 2007; Pulido *et al.* 2018). Stress and fatigue in ruminants are closely linked (Knowles & Warriss 2000; Ferguson & Warner 2008). Any condition or combination of conditions that imposes a coping burden on the animal will use up the animal’s energy, leaving less energy available to stave off fatigue which ultimately lessens its welfare (Cockram 2004, 2007). Fatigue can also lead to economic loss as fatigued animals have impaired welfare (reduced ability to cope with their environment) which, in turn, results in diminished productivity, increased susceptibility to disease, higher mortality, and lower meat quality (Llonch *et al.* 2015; Hemsworth *et al.* 2019). Stress (which is closely linked to fatigue) and the resulting decreased welfare can lead to physiological changes associated with reduced meat quality (Hemsworth *et al.* 2019).

The present project aimed to add to the body of knowledge on the topic of sheep fatigue by collecting and critically assessing stakeholder views as regards sheep fatigue, with a focus on transport, including on issues such as understanding and ability to identify fatigue, complications of doing so in transport conditions, and interplay between fatigue and other transport- and animal-specific factors. As noted, the project focused on the EU.

### Regulation

Council Regulation (EC) No 1/2005 (2005) (which is implemented in the UK through a number of regulations and remains in force) protects livestock species, including sheep, during live transport within the EU. Under Regulation 1/2005, an animal can be transported only if it is fit for its intended journey, and physiological weakness (such as that associated with fatigue) would render an animal unfit. Regulation 1/2005 does not explain how to identify fatigue. Related guidance focuses on “severe fatigue or exhaustion”, signs of which include “chin or limbs resting at partitions or troughs, closed eyes, high drive to rest in recumbent position”, “general lethargy, apathy, lack of reaction” and “inability/reluctance to rise” (Consortium of the Animal Transport Guides Project 2018; pp 13, 45). To identify fatigue that has not yet become exhaustion, one is instructed to assess the animal’s “posture and resting behaviour” (Consortium of the Animal Transport Guides Project 2018; p 12). Issued in connection with the ongoing revision of Regulation 1/2005, the European Food Safety Authority [EFSA] Opinion (Nielsen *et al.* 2022) explains that scientific research on fatigue is scarce. Click or tap here to enter text.

On December 7, 2023, the EU unveiled a suite of proposed changes to Regulation 1/2005 (European Commission 2023). Unlike Regulation 1/2005, Article 4(2)(a) of these proposed changes mentions fatigue among animal welfare (“AW”) issues to be reduced by minimising the duration of the journey. Several other aspects relevant to fatigue bear mention. Article 4(2)(i) requires that transporters offer rest in a way that meets animals’ physiological needs. Article 28 specifies that transport for slaughter should be carried out only in short journeys (generally not exceeding 9 h; Article 3[12]). These changes, together with the increase in space allowance per the EFSA Opinion in Nielsen *et al.* (2022) to allow rest on the vehicle (Preamble 30) and shortening of the overall permissible journey time (Article 27), may incrementally improve animals’ ability to rest and potentially decrease fatigue. Unfortunately, sea transport, which has been found to be fatiguing (Santurtun *et al.* 2015; Navarro *et al.* 2017), does not count toward journey time limits (Preamble 41). Therefore, animals will continue to be exported by sea via potentially very long journeys (Eurogroup for Animals & CWF 2023).

### Scientific knowledge

Although scientific understanding of fatigue in sheep remains limited (with the issue not having been studied), stress, fear, and anxiety have been found to cause fatigue in humans (Boksem & Tops 2008; Sabaner *et al.* 2022). By analogy, stress, fear, and anxiety associated with transport (Wemelsfelder & Farish 2004; Hemsworth *et al.* 2019) could result in mental fatigue in sheep.

A related concept of sensory overload bears mention (Baker 1984). Each individual can integrate sensory inputs up to a certain limit, and when those coping limits are exceeded either due to the intensity of stimulation or due to a simultaneous experience of several intense or novel stimuli, the individual is said to be experiencing sensory overload and may suffer from fear, anxiety, and other disorders as a result (Baker 1984). Fatigue (along with stress) is a recognised cause of sensory overload in humans (Scheydt *et al.* 2017).

Sensory overload has also been studied in primates (e.g. Andersen *et al.* 2014) and rats (Stevens *et al.* 1993) and has been hypothesised to exist in other species (Stevens & Ruxton 2014). No research specific to sensory overload in sheep has been identified. The EFSA Opinion in Nielsen *et al.* (2022) mentions it a number of times but does not cite scientific evidence, which is not surprising given that this is a novel area of research. Nevertheless, sheep can experience negative affective states (Doyle 2017) and find transport highly aversive (Parrott *et al.* 1994). Sheep olfaction may be as sensitive as that of dogs; there is also evidence that sheep are sensitive to sound (Kendrick 2008; Weeks 2008). Sheep may therefore be stressed and disturbed not only by smells and noises detectible by humans but also by those that are not (Kendrick 2008; Weeks 2008). The combination and intensity of multiple stressful stimuli associated with transport could result in sheep experiencing sensory overload.

#### Behavioural signs

The conventional view of sheep as stoic prey animals which do not readily show signs of problems may reflect our lack of understanding, rather than any inherent characteristics sheep may possess (Wemelsfelder & Farish 2004; Doyle 2017). While studies have identified several fatigue-related behaviours (Table 1), including lying down, some of these can also indicate other issues or the absence of a problem (Hart 1988; Hall *et al.* 1998; Cockram & Mitchell 1999; Cockram *et al.* 1999; Cockram 2004, 2007; Bøe *et al.* 2006; Nielsen *et al.* 2011, 2022; Santurtun *et al.* 2015; Navarro *et al.* 2017, 2018). Studies referring to changes in typical behaviour or motivation suggest that prior familiarity with the individual animals (rarely possible in commercial transport) could help identify fatigue (Cockram *et al.* 2012; Llonch *et al* 2015).

**Table 1.** Behaviours indicative of fatigue and their potential ambiguity

Behaviour	Relevance to Fatigue?	Relevance to Another Issue?	Can Indicate That There is No Issue?
Inability to get up	Inability to rise can indicate fatigue (Nielsen *et al.* 2022)	Inability to rise can indicate lameness or high stocking density + slippery floor (leading to the animal being pushed down and being unable to rise) (Nielsen *et al.* 2022)	
Lying behaviour - general	Lying can indicate fatigue (Nielsen *et al.* 2022) Absence of lying cannot be interpreted as indicating the absence of fatigue where there is no room to lie down, as is typically the case in commercial transport (Hall *et al.* 1998) Lying in less-preferred areas (e.g. highly trafficked) can indicate fatigue (Bøe *et al.* 2006)	Lying can indicate another issue – see above (Nielsen *et al.* 2022)	Lying can indicate normal resting (Nielsen *et al.* 2011) Need to look at all the facts and circumstances (Cockram & Mitchell 1999) No clear relationship between lying time and fatigue (Cockram *et al.* 1999)
Lying behaviour - position	Lying position could shed light on whether the animal is resting or exhausted (Hall *et al.* 1998)		A sternal position with the legs tucked under may indicate a calm and relaxed state (Wemelsfelder & Farish 2004), particularly if the animal is ruminating (Cockram & Mitchell 1999)
Lying behaviour – increased lying time	Increased lying can indicate muscle fatigue (Cockram 2007)	Increased lying can indicate sickness (Hart 1988)	Increased lying can indicate habituation to the transport environment (Cockram 2007)
Lying behaviour – decreased lying time	Decreased lying can indicate fatigue: indicates stress (Nielsen *et al.* 2011) which is linked to fatigue		
Panting	Panting can indicate fatigue (Cockram 2004)	Panting can indicate heat stress or psychological stress (Cockram 2004)	
Rumination	If decreased, can indicate fatigue: indicates stress (Nielsen *et al.* 2011) which is linked to fatigue	If decreased – reaction to vehicle movement (Santurtun *et al.* 2015); reaction to stress (Cockram 2004) Rumination can also decrease as a result of pain (Ibrahim *et al.* 2018), and is affected by a number of other factors beyond the scope of this paper	
Sickness behaviour (e.g. dull and listless appearance, slow movement, apathy)	Sickness behaviour can indicate fatigue (Nielsen *et al.* 2011; Rabitsch 2023)	Sickness behaviour can indicate disease (Nielsen *et al.* 2011) Lack of responsiveness to approach or touch can indicate a problem (Phythian *et al.* 2013)	
Stepping motion	Stepping motion can indicate muscle fatigue (Santurtun *et al.* 2015)	Stepping motion can indicate negative emotions or stress (Navarro *et al.* 2017)	
Post-transport behaviour	Some studies suggest that behaviour on arrival (e.g. activity prioritisation and time to recovery of any weight loss and latency to return to normal eating, drinking, and resting patterns, may shed light on the stress and fatigue experienced during transport (Cockram *et al.* 1999)	Many factors could alter the animal’s normal eating, drinking, and resting patterns, care should be taken in drawing firm conclusions as to their meaning (Cockram & Mitchell 1999)	Caution should be exercised before concluding that sheep were not fatigued where they did not lie down soon after transport; many other factors (including availability of feed and water) could have influenced lying behaviour (Cockram *et al.* 1999)

#### Physiological signs

Physiological measures of fatigue (e.g. blood plasma concentration of cortisol or creatine kinase) that can reflect mobilisation or depletion of energy reserves and thus underpin muscular fatigue and metabolic exhaustion have been used in research (Broom *et al.* 1996; Cockram *et al.* 2012). However, they are not practical in commercial setting (Herman 2022). Values can be affected by the timing and the process of obtaining the sample and, even if a change is detected, its significance for AW may not be clear (Wickham *et al.* 2015).

Care should be taken not to draw strong conclusions from physiological measures alone. While behaviour has been reported to correlate with physiological changes in the context of transport (Wickham *et al.* 2012), this is not always the case. The animal may be exhibiting behavioural indicators of distress but not showing physiological changes indicative of distress (Cockram 2004). Marked differences in response to the same stimulus can exist animal-to-animal (Hemsworth *et al.* 2019) and, for the same animal, situation-to-situation (Cockram 2004). Some physiological parameters have been found to vary seasonally (Baldock & Sibly 1990) and by breed (Hall & Bradshaw 1998). Certain hormonal responses (e.g. prolactin levels) vary for reasons that are not well understood; changes in others (e.g. plasma cortisol, haematocrit) may be influenced by their initial levels or dietary changes (Broom *et al.* 1996).

#### Animal and transport conditions

The animal’s age, health, and condition all affect how it experiences and expresses fatigue (Nielsen *et al.* 2011; Messori *et al.* 2015a; Hemsworth *et al.* 2019). Other factors, such as whether it is shorn or unshorn (DG Health & Food Safety 2020), its breed (Hall *et al.* 1998), temperament (Collins *et al.* 2018), and personality (Koolhaas 2008) are also relevant. The animal’s pre-transport history (such as rearing environment and, more immediately, transit through markets and food and water deprivation) affects its ability to cope with stressors (Kim *et al.* 1994; Hall & Bradshaw 1998; Nielsen *et al.* 2011; Collins *et al.* 2018) and therefore its fatigue threshold. It is important to assess the effect of transport from the point of view of sheep, rather than assuming that they would react as humans would (Hall & Bradshaw 1998).

Habituation to transport through repeated exposure is theoretically possible (Cockram *et al.* 2000; Wickham *et al.* 2012; Messori *et al.* 2015a). For example, Wickham *et al.* (2012) claim to have achieved habituation by repeatedly transporting sheep (90-min trips on seven of eight consecutive days); the study then compared the animals’ physiological and behavioural responses on first exposure to transport with those after presumed habituation. However, sheep are not transported often enough to consider habituation feasible in a commercial transport context (Nielsen *et al.* 2011).

Both journey time (including number of stops) and quality will influence how a sheep is affected by transport (Nielsen *et al.* 2011). Journey quality is a term commonly used to refer to the journey conditions such as (a) driving style (e.g. smoothness of braking or acceleration) and speed; (b) vehicle type (e.g. open or closed) and quality (e.g. suspension); (c) road type (e.g. curved/straight) and nature (e.g. paved or unpaved); (d) climate within the vehicle (temperature, humidity, air quality) or outside if relevant; (e) social environment (e.g. number of animals and stocking density, whether the animals are familiar with one another); (f) access to feed and water; (g) lighting; (h) type and quality of bedding; and (i) headroom, to name a few (Nielsen *et al.* 2011).

Factors set out in items (a)–(c), along with the number of stops, determine the degree of motion stress experienced by the animals (Cockram *et al.* 2004; Cockram 2007; Nielsen *et al.* 2011). Unpredictable or irregular motion is more stressful than predictable or regular motion (Ruiz-de-la-Torre *et al.* 2001; Navarro *et al.* 2018) and both can cause fatigue (Cockram *et al.* 2012). As the journey progresses, motion stress and the resulting fatigue impair sheep’s ability to adapt to vehicle movement, increasing the risk of injury (Cockram *et al*. 2012; Santurtun *et al.* 2015). In response to motion or acceleration, sheep (transported standing) seek to resist postural instability, compensating for postural destabilisation by muscle contraction in response to imposed forces (Jones *et al.* 2010; Santurtun & Phillips 2015). Over time, the energy exertion inherent in muscle contraction can contribute to fatigue.

Pre-transport management and loading and unloading experience can also be considered part of the overall journey quality (Nielsen *et al.* 2011). Studies concluding that sheep find loading and unloading more stressful than the journey itself (e.g. Broom *et al.* 1996) are often cited in favour of extending journey times without letting the animals off the vehicle (Messori *et al.* 2015a). While loading and unloading can be stressful and tiring, Messori *et al.* (2015a) found that AW grounds did not justify avoiding unloading sheep for a rest break. Although short journeys in bad conditions can be fatiguing, generally, the longer the journey, the more susceptible the animal is to fatigue (Nielsen *et al.* 2011). This is due to the cumulative nature of a number of stressors affecting sheep in transit, several of which cannot be reduced except by ending the journey (Nielsen *et al.* 2011).

Rest stops in long-distance sheep transport are mandated as follows. Sheep can be transported for two blocks of 14 h separated by 1-h break on the vehicle (Regulation 1/2005). If further transport is required after this period, the animals must be unloaded into a rest area for 24 h and provided with feed and water of a suitable type and quantity, and an opportunity to recover before the journey continues (Regulation 1/2005). Concerns have been raised about the AW impact of the 1-h stop (without unloading) required after 14 h of road transport (Cockram *et al.* 1997; Cockram & Mitchell 1999; Nielsen *et al.* 2022). The 24-h unloading of animals into a rest area required after two 14-h blocks of transport separated by the 1-h break on the vehicle may be sufficient to allow recovery from fatigue, but only in sufficiently good conditions (Cockram & Mitchell 1999) at least as to food, water, and rest opportunities, as mandated by Regulation 1/2005.

Stocking density and overhead space, together with environmental factors, and quality and availability and distribution of ventilation can significantly affect air temperature, humidity, and overall quality (Nielsen *et al.* 2022), which are all relevant to the animal’s coping threshold, stress, and fatigue. Sheep find space restriction aversive (Navarro *et al.* 2018) and require more energy to cope with higher stocking density during transport (Akin *et al.* 2018). As the body of the animal uses its energy resources, their depletion contributes to fatigue (Sahlin *et al.* 1998). Being herd animals, sheep prefer to synchronise their behaviour (Jørgensen *et al.* 2009). High stocking density, typical of commercial transport, particularly when coupled with rough driving and difficult roads, has been found to hinder attempts to synchronise lying behaviour (Messori *et al.* 2015a) and cause other behavioural and physiological signs of stress, including reduced lying and rumination, decreased ability to balance while standing, increased aggression, and changes in heart rate variability (Cockram *et al.* 2004; Cockram 2007; Jørgensen *et al.* 2009; Nielsen *et al.* 2011; Navarro *et al.* 2018).

Unavailability of food and water can deplete the body’s energy resources, leading to fatigue (as noted above, this depletion may be detectable via physiological markers not practical in transport setting). Drinking in transit is complicated as nipple drinkers provided during commercial transport are novel to most sheep and difficult to access due to overcrowding and fear of unfamiliar conspecifics (Fisher & Matthews 2001; Nielsen *et al.* 2022). There have been studies suggesting that sheep can handle water deprivation better than other farm animal species (Cockram 2007), but at least some raise bias (Fisher *et al.* 2010) and study design concerns (Messori *et al.* 2015a). Fisher *et al.* (2010) is a potentially biased study because it was sponsored by the Australian sheep industry. It deemed sheep welfare acceptable during journeys of up to 48 h without food and water and observed no marked fatigue on arrival (Fisher *et al.* 2010). Messori *et al.* (2015a) raises study design concerns as they had water in buckets available on the truck, which would not happen in commercial transport. Further, to the extent that the results in Messori *et al.* (2015a) are based on lack of changes in haematocrit, these results are questionable because haematocrit may not be sufficiently sensitive to detect even substantial fluid shifts within the body (Painter *et al.* 1947). As sheep prioritise eating and drinking over rest, if they are unable to access food and water, they will not rest (Cockram *et al.* 1997). Even if resources are available, psychological stress can reduce eating and drinking (Cockram *et al.* 2000) and impair rest (Messori *et al.* 2015a).

One study of sheep fatigue during transport, constrained by ethical considerations, failed to find any physiological or behavioural signs of fatigue in sheep exposed to extended periods of gentle treadmill walking (Cockram *et al.* 2012). Although the study did not involve transport, treadmill walking has been used to induce fatigue and the study sought to develop a reproducible, reliable model of sheep fatigue response to low-intensity, long-duration exercise considered similar to transport (Cockram *et al.* 2012). Nevertheless, this study’s practical significance is limited, except with regard to clarifying what exertion type does not appear to fatigue sheep.

#### Fatigue in proposed sheep welfare assessment frameworks

Given the limited body of research on the issue of fatigue, it is perhaps not surprising that it appears only peripherally in proposed sheep welfare assessment frameworks (Cockram & Mitchell 1999; Phythian *et al.* 2013; Llonch *et al.* 2015; Messori *et al.* 2015b; Wickham *et al.* 2015; Willis *et al.* 2021). Most frameworks proposed for commercial use (listed in Table 2) pertain to assessments during or post-transport; only one is focused on on-farm welfare assessment.

**Table 2.** Fatigue in frameworks for sheep welfare assessment

Study	Reference to Fatigue
Phythian *et al.* (2013) – propose an on-farm welfare assessment framework for sheep	Does not discuss fatigue, but may imply it as the possible cause of dull, non-responsive, or recumbent behaviour
Wickham *et al.* (2015) – propose using QBA to assess sheep welfare during transport	Notes that observers described some animals as “tired/passive/terrified”; the researchers observed that QBA could be used to identify less obvious affective states such as tiredness
Messori *et al.* (2015b) – propose a welfare assessment for sheep after long transport	Includes severe exhaustion as a welfare assessment measure, fatigue is either not mentioned or is referred to only in passing
Llonch *et al.* (2015) – propose a welfare assessment framework for sheep for use on farm, at markets, and in transport	Mentions fatigue briefly in discussing sickness (i.e. fatigue being a potential indicator that the animal is unwell) and includes normal behaviour as a criterion, potentially implying that fatigued behaviour is not normal
Willis *et al.* (2021) – propose a welfare assessment framework for sheep during sea transport	Includes a “lethargic” category, encompassing “[l]acking interest, dispirited, apathetic, slow moving, listless, [and] dull” behaviour, which could suggest that the animal is fatigued, and three alertness-related categories (“active,” “alert”, and “inquisitive”), encompassing behaviours that could suggest the absence of fatigue (p 4) Notes the importance of sufficient rest, but does not expressly mention fatigue

### Relevance of stakeholder perceptions

Understanding the opinions and motivations of individuals affected by laws or regulations relating to AW (such as farmers, hauliers, or consumers) is important for the development of such rules to maximise their effectiveness and increase the likelihood of compliance (Vaarst 2003; Molnár & Fraser 2020; Kuo & von Keyserlingk 2023). Stakeholder views, collected through qualitative methods such as interviews, have been considered in several studies relating to AW (Horseman *et al.* 2014; Palczynski *et al.* 2020; Molnár & Fraser 2021). A comprehensive overview of such studies is beyond the scope of this project but, by way of example, farmers’ views of AW have been found to affect how they treat animals, thereby directly impacting productivity and other welfare indicators (Jääskeläinen *et al.* 2014). They can also affect the effectiveness and implementation of welfare assessment schemes, as suggested by another study that explored farmer views regarding on-farm welfare assessments of cattle, pigs, and mink (Vaarst 2003). Understanding stakeholder perspectives can contribute to bridging the divide between conflicting views as well as identify solutions that could ultimately improve AW (Humble *et al.* 2021; Schuppli *et al.* 2023).

Views of stakeholders who work with and within the sheep transport industry with regard to sheep welfare during transport, with a focus on fatigue, have not been previously considered. Accordingly, a qualitative study was planned to collect stakeholder views via semi-structured interviews and apply reflexive thematic analysis ‘TA’ to the resulting data. The research aim was to contribute to the discourse around and understanding of sheep fatigue during transport by gaining insight into and critically assessing knowledgeable-stakeholder perceptions around this issue, with the ultimate goal being to generate new knowledge that could ultimately help improve sheep welfare. The focus of the study was on the EU, with which all but one interviewee had strong ties.

## Materials and methods

### Ethical approval

The study was approved by the University of Edinburgh’s Human Ethical Review Committee (approval 2022-116).

### Interviews

A semi-structured interview approach was followed (Turner 2010). This entailed preparing an interview guide (the final version can be seen in Table S1 in the Supplementary material) organised around research topics (Leech 2002). The focus was on the understanding of sheep fatigue and interplay between fatigue and other transport circumstances. While the research topics remained broadly similar across all interviews, using the semi-structured method and a guide (rather than a detailed script, with a strictly observed set of questions to be asked in the same order and in the same words without variation) allowed for the possibility of covering related topics that arose during discussion, leading to a more natural and dynamic conversation. This method was selected due to its balance between structure, enabling interviewer control and relatively uniform data collection, and flexibility, allowing the interviewer to follow-up on specific points (Turner 2010).

The interview guide reflects the comments of two AW scientists. It was not pilot-tested with these scientists since they were unfamiliar with sheep fatigue. While pilots can help refine the approach (Kallio *et al.* 2016), no pilot was conducted due to the nature of the questions (requiring specialised knowledge) and the limited initial number of prospective participants (who, had they participated in the pilot, would have had to be excluded from participation in the project) (Chenail 2016).

The interview guide was refined after the first few interviews to allow for sufficient time for a fuller and more complete discussion of stakeholder perceptions of fatigue. Certain topics (e.g. fitness for transport) were removed, others added (e.g. questions relating to the possible significance of lying position), and yet others only discussed if time permitted/if brought up by participants (e.g. sufficiency of EU regulation with regard to fatigue).

KC (the interviewer) did not personally know any participant prior to the project and the initial set of candidates was drawn from the researchers’ network, bearing in mind the following inclusion criteria: (1) professional knowledge of sheep as species (being familiar with sheep transport is a plus, but not a pre-requisite) or, if focus on other species, background in EU-based sheep welfare related projects; and (2) sufficient knowledge of English. These individuals were contacted via email, enclosing a description of the project including its scope and purpose and informed consent forms. Each person that had expressed an interest in participating had an opportunity to ask questions (and refuse participation at any point during the interview); written consent forms were obtained from all participants.

Of the 60 individuals contacted in connection with the project, 30 did not reply, 12 declined, and 18 agreed to participate and were subsequently interviewed. Of the final 18 participants, six were recruited through the researchers’ network; the remaining 12 participants were recruited through snowball sampling. Snowball sampling, often used in qualitative research, entails starting with an initial interviewee set identified through the researchers’ network, then expanding it to include individuals recommended by the initial interviewees that also fit the target criteria (Parker *et al.* 2019). This process can continue to include those suggested by the individuals recommended by the initial interviewee set and so on (Parker *et al.* 2019). In the present project, referrals were also solicited from individuals who declined participation. Such referrals by non-participants resulted in the recruitment of five interviewees.

This approach effectively identified a number of experts across several stakeholder groups and in geographies of interest. Interviews started in October 2022 and ended in March 2023. Eighteen individuals from nine countries were interviewed. Each remote, video-recorded interview lasted between 45 and 75 min. With the exception of the first three interviews recorded using Zoom®, the interviews were recorded using Microsoft® Teams. The target of identifying stakeholders in countries with significant live sheep transport (Greece, Italy, Romania, and Spain) was met as to Spain and Italy. Interviewee breakdown by country of residence was as follows: Australia (3), Canada (1), Denmark (1), Germany (1), Italy (1), the Netherlands (2), Singapore (1), Spain (1), the UK (7). The UK predominates, with approximately 40% of the interviewees residing there. This may be due to the language of the project, the nature of the recruitment process, or the fact that sheep are commonly raised and transported within the UK.

The inclusion of five individuals based outside the EU/UK does not undermine the study’s EU/UK focus. Without disclosing identifying details, four of the five individuals based outside the EU/UK had strong EU/UK connection through being raised and/or educated in the EU or the UK or having been directly involved in at least one major EU-based project pertaining to sheep welfare. Only one individual based outside the EU/UK did not have such a nexus but was aware of relevant EU science and regulatory developments.

Interviewee stakeholder affiliations are as follows:

- Governmental authority (current or former government employee) (4);
- Non-governmental organisation (‘NGO’) focused on AW (4);
- AW scientist (employee of a research institution and focused on AW-related research and/or engagement with industry on AW topics) (4); and
- Industry (representative of industry association, transporter, or industry consultant) (6).

In each case, the views expressed were those of the stakeholder in their individual capacity, not on behalf of a particular organisation. In reporting the results, to protect interviewee confidentiality, participants were randomly assigned code names corresponding to breeds of domestic sheep.

Veterinarians account for around 40% of the interview set and are present across all categories except AW scientists. One-third of the interviewees farmed sheep at the time of the interview or had done so previously. Five of these individuals fall within the industry category, with one falling within the AW scientist category. Some interviewees also specialise in other livestock species in addition to sheep. It was decided to interview three individuals primarily focused on other species, as their knowledge could add variety and perspective to the analysis. The value of their perspective is evidenced by the fact that all three have been involved in major EU-based projects relating to sheep welfare.

Individuals with a stronger focus on AW might have been more willing to participate and may therefore be overrepresented in the dataset (Bethlehem 2010). Further, women, accounting for 40% of the interviewees, reportedly exhibit a higher degree of sensitivity than men on AW-related topics (Pulido *et al.* 2018). These characteristics of the interviewees and the researcher interest in AW might have affected the interpretation of the data. Interviewer biases and verbal and non-verbal cues may also have influenced the process (Turner 2010). Given the nature of the analysis (reflexive TA), however, the interviewer forms a valuable part of the analysis and subjectivity, when openly acknowledged, can bring richness and depth to the analysis (Braun & Clarke 2021a).

The following factors that may have influenced the interview process and the analysis bear mention in relation to the interviewer, KC. KC, a middle-aged, female lawyer with a long-standing interest in AW, has never had close interaction with sheep. In late 2023, KC obtained an MSc degree in International Animal Welfare, Ethics, and Law from the Royal (Dick) School of Veterinary Studies at the University of Edinburgh.

While this project was KC’s first foray into reflexive TA, as an attorney with 20 years of experience, she has conducted many interviews and has experience with critical analysis of interview data. The project described in this paper formed part of her dissertation thesis toward the MSc degree. KC’s long-standing pro-AW sentiment could have influenced not only the interviews but also the interpretation of the data. While neutral and friendly demeanour was maintained throughout the interviews, KC’s reservations about certain statements made by industry stakeholders (e.g. to the effect that sheep are virtually indestructible) are acknowledged. MM and FL are AW scientists with decades of experience in research and teaching. Both have researched sheep and animal transport, and MM is widely considered an expert in the latter. Although not directly involved in the interviews, MM and FL oversaw all aspects of the project as KC’s dissertation supervisors.

Data saturation, denoting a point at which no new information is identified (Sargeant 2012), is a concept used in some types of qualitative research to indicate sufficient sample size. It was, however, not appropriate for use here as it is not congruent with the “values and assumptions” of reflexive TA (Braun & Clarke 2021b). Braun and Clarke (2021b) advocate, instead, for the concept of “information power”, under which the more information relevant to the study at hand a sample contains, the fewer participants are needed. Information power is thus directly related to the scope of the study: in a narrowly scoped study such as the project at hand, sufficient information power can be achieved with fewer participants (Malterud *et al.* 2016).

The project’s focus is on the EU perspective. The differences between the EU and UK regimes are not noted as: (1) the UK only recently left the EU and much of available data still include the UK as part of the EU; and (2) none of the UK interviewees highlighted divergences between EU and UK legislation.

### Reflexive TA

This study applied reflexive TA developed by Braun and Clarke (2022). Reflexive TA recognises the researcher’s active, interpretive role in meaning-creation; there is no expectation of repeatability or neutrality, no drive to distance the researcher from the results or denounce bias (Braun & Clarke 2021a). As noted, as long as they are openly acknowledged, researcher biases form an acceptable and desirable part of the subjective, deep, and interpretive process (Braun & Clarke 2021a). Consistent with best practices, Table S2 in the Supplementary material situates the approach used in this study as to four key aspects of analytical framework.

Reflexive TA entails six iterative phases, with the researcher repeatedly re-visiting earlier phases to refine the analysis (see Table S3 in the Supplementary material). Consistent with best practices set out in Braun and Clarke (2022), the explanations reflect what was actually done (rather than restating the generic phases set out in Braun and Clarke’s guidance).

Reflexive TA is not premised on a quantitative approach to theme development: a theme is a pattern of shared meaning developed from multiple participants’ data, but frequency of appearance does not equal importance (Braun & Clarke 2022). A prevalent theme can be uninteresting for the analysis; conversely, a theme developed from only a few participants’ data can be novel and important (Braun & Clarke 2022). Accordingly, no theme prevalence quantification is used in this paper.

## Results and Discussion

The research questions focused on stakeholder views regarding sheep fatigue, its interplay with transport circumstances in EU context, and its implications for sheep welfare. Applying reflexive TA to address these questions resulted in the development of the following themes and subthemes, unified by the overarching theme “Human ‘spectacles’ are biased”:

1. “Let’s anthropomorphise it a little bit”. This theme includes the subtheme “They are tired of being nervous”;
2. “We think that they’re like we are and they’re not”. This theme includes the subthemes: “Not all stress is the end of the world” and They are “resilient until they’re not”;
3. See the whole animal; and
4. Fatigue “never comes up”.

**Figure 1.**
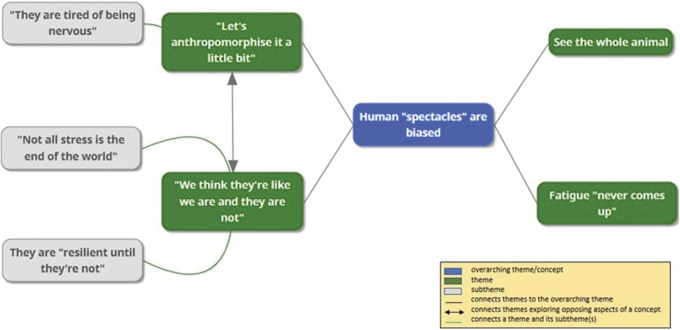
Thematic map

The quotation marks around names signify that the phrase is a participant quote. These phrases are also reproduced below, in fuller context in discussion of the relevant themes and sub-themes. The thematic map setting out the themes and subthemes developed in the course of this project is reproduced at Figure 1.

The following background is relevant to the first two themes, which cover different aspects of anthropomorphism. Anthropomorphism can be defined as attributing human emotions, motivations, and “mental states … to nonhuman animals” (Serpell 2003; p 83). An extension of human ability to infer what other humans may think or feel, it is a natural way of making sense of the world by drawing conclusions about other animals’ experiences based on our own (Serpell 2003; Dacey 2017). Long dismissed as unscientific (Sober 2006), anthropomorphism is more accepted today (Wood 2019).

Anthropomorphism can help us understand things, convey information, and influence others (Wood 2019). People are more likely to anthropomorphise to close a “knowledge gap” (Wood 2019; p. 29) (i.e. in our understanding of sheep fatigue). The capability anthropomorphism has both to communicate and persuade has been harnessed to help influence public opinion on issues such as conservation (Tam 2015) and captive animal welfare (Rowley & Johnson 2018).

Anthropocentric anthropomorphism uses a “‘human’ point of reference” (Bouma *et al.* 2022; p 2) to interpret animal behaviour, attributing to animals human thoughts and qualities that have little relationship to what is known about the animal (as can be seen in fairytales) (de Waal 1999; Rowley & Johnson 2018). By contrast, animalcentric anthropomorphism focuses on the animal’s perspective: it “draw[s] parallels” between human and animal experiences “without denying possible differences” and considering the relevant species’ history and habits (de Waal 1999; p 264). An example might be the description by Orkhon (AW scientist) of the behaviour of one of their sheep following stillbirth. Orkhon draws parallels between the experience of grief and loss (also shared by humans) but notes circumstances and behaviour specific to sheep (trying to rouse the dead lambs): “*[S]he walked around the field, making a noise that sounded like she was crying. … [T]he patterns of her breathing and bleating were coming out like someone that was struggling to get their air because you’re emotionally upset.”*

Heuristic anthropomorphism (mainly an offshoot of animalcentric anthropomorphism) supports “scientific exploration” in a way similar to “intuition and informed ‘hunches’” by helping frame research and generate hypotheses for testing (de Waal 1999; p 270). Both anthropomorphism and its opposite, dubbed anthropodenial by de Waal (1999), can lead to correct or erroneous conclusions.

### “Let’s anthropomorphise it a little bit”

The focus of this theme is on anthropomorphism as an intuitive and perhaps inevitable way for humans to understand the world: “without reference to human experience … there is no human understanding” (de Waal 1999; p 263, citing Midgley 1978). Most participants made assumptions as to how sheep would experience fatigue (and transport) based on their own actual or imagined experience of similar circumstances, hypothesising about potential similarities between sheep and human experiences while keeping sheep perspective in mind:

*Let’s anthropomorphise it a little bit … [F]atigue … is a really quite a complex thing … I can come home from work and be fatigued, but I could … hide it and pretend not to be, while also wanting to die slowly in a corner. But if you had guests come around … you have to do whatever you need to do* [to] *get on with it. So, it’s no different with animals* [Edilbay, industry].

*I would compare it to yourself. … If you’re in a very hot bus and there’s not much fresh air, there’s ammonia building up because the toilet is overflowing, for example, and it’s hot, [t]hen you feel bad, you feel exhausted and heavy … and on a truck, you should not be sleepy and weak because you need to defend your position, you need to remain standing* [Marwari, NGO].

*[I]f there is insufficient space for them to move around one another, and lie down sufficiently … you just see a lot of posture changing and that, over time … must be tiring[.] … I am anthropomorphising here, but as another mammalian species … if you are having to stand up and change posture all the time … throughout a journey, that’s tiring* [Orkhon, AW scientist].

The dearth of scientific information on sheep fatigue and the fact that fatigue also affects humans (as opposed to being a sheep-specific issue) may have made it even more natural for participants to anthropomorphise (Lockwood 1986), resorting to the best source of information available – themselves. Participants may have also used anthropomorphic comparisons to better convey ideas.

Rather than trying to avoid anthropomorphism or using it without reflection, we could use it in a critical, reflexive, and considered way to drive better understanding of sheep fatigue. Where there is a “potential for analogous experiences” and a “good understanding of the animal’s ecological, evolutionary, and individual history” (Lockwood 1986; p 193) (as is the case with sheep fatigue, with the exception of individual history in a commercial transport context) we could anthropomorphise to generate hypotheses, the investigation of which could lead to valuable insights regarding sheep fatigue (de Waal 1999). Further, anthropomorphism could be used in the context of live sheep transport to drive a further shift of public opinion (and policy) on whether live animal transport should continue in its current form, or at all (Rowley & Johnson 2018). Any advocacy materials would need to account for audience demographics, as receptiveness to anthropomorphism varies (Tam 2015).

#### “They are tired of being nervous”

This subtheme focuses on the possibility of mental fatigue in sheep, a topic on which conscious animalcentric anthropomorphism could guide further inquiry. While a few interviewees doubted whether sheep might experience mental fatigue or saw no practical way to opine on it given the lack of knowledge, some suggested that, like humans, sheep can experience mental fatigue or sensory overload (Baker 1984). For example, Edilbay (industry) noted: *“I have no doubt that there is mental fatigue …‘cause they’re either with a new group or they’re again in a new environment and therefore that is stressful. I put … all that together … within … stress* [and] *those mental stressors do relate to fatigue.”*

This Darwinian idea of “a continuity of mental experiences” (Lockwood 1986; p 186) is not present in the literature on sheep fatigue. Rather, the focus is on muscle fatigue, and even “central” fatigue is seemingly described as purely a physical phenomenon (Cockram *et al.* 2012). By contrast, mental fatigue has been considered in rodents (Bai *et al.* 2021; Niepoetter *et al.* 2022) and non-human primates (Baker *et al.* 2023).

One example of a comparison between sheep and humans is the following description by Orkhon (AW scientist) of their flock of sheep after a stressful encounter with a dog as being “*mentally tired … and then hypervigilant … which was also more tiring*”. Additional examples are:

*[B]y analogy with humans, then, the constant stressors which animals are exposed to by the time they get to the importing country would lead to an element of mental fatigue. And you can almost see it on some of the sheep[:] … they would lie down in a corner and they’ll crook their head back to their thorax; they’ve given up. … Everything has changed for them, and …there’s likely mental fatigue, which will accelerate the physical fatigue* [Polwarth, AW scientist].

*And … mental fatigue. They are tired of being nervous. They’re tired of being in a novel situation, not knowing … imagine how stressful that is for an animal who’s used to getting water always in the left corner of its pen and a water system where you have to press the lever and water comes out. And now, all of a sudden, they don’t even know where the water is, and it’s a nipple, …and it’s two meters this way, and I have to go past a dominant animal. It must be horrible* [Marwari, NGO].

Note how, toward the end of their answer, Marwari switches to first person, implicitly placing themselves in the position of a sheep and empathising with it.

An aspect of this subtheme is the idea that sheep may experience sensory overload, which is associated with fatigue and stress (Scheydt *et al.* 2017). Nielsen *et al.* (2022) note the possibility that sheep may experience sensory overload, but do not cite any support. To the extent that statements might have been implicitly based on interviewees’ own experience of being subjected to multiple stimuli at the same time, these would be, in essence, anthropomorphic. Gaddi’s use of the word “you” in the following description suggests that they are placing themselves in the sheep’s shoes: “*I wouldn’t be surprised at, also mentally, if you have overwhelming noises and strange environment, that can contribute to fatigue.”* (Further, absence of studies on this issue may have made it easier and more natural to resort to anthropomorphism, as noted above [Lockwood 1986]).

While we can hypothesise about the potential similarities between humans and sheep on this issue, we cannot fully appreciate the extent to which sheep may be affected by the combination of stressors and stimuli of commercial transport. As Bibrik (NGO) notes: “*it’s very naïve of us to think that we actually will ever understand how they experience the world. A lot of them have senses way past our capacity[.]*” For example, sheep and humans hear different sound frequencies (Weeks 2008) and therefore sheep may experience noise associated with transport in ways humans cannot understand. This subtheme highlights an important area of further inquiry, which seems to have been overlooked so far, and which may be valuable to improving sheep welfare during transport.

### “We think that they’re like we are and they’re not”

The focus of this theme is on the dangers of anthropocentric anthropomorphism, which can seep into practices and regulations, leading to ineffective animal management and negative AW consequences (Serpell 2003; Bouma *et al.* 2022). We might not realise that something is an issue and therefore not attempt to address it, or we may attempt to solve a correctly identified problem in an inappropriate way (Hall & Bradshaw 1998). Most respondents suggested that our thinking around sheep transport, the context within which this project examines fatigue, may be anthropocentric:

*I don’t know whether they would actually eat if you got them off a trailer, and just put them in an area. … Like rushing to the service station to get a cup of coffee: we would know we’ve got 10 minutes to do it, but they wouldn’t have a perception of that* [Alai, industry].

*[W]e think that they’re like we are and they’re not. …They’re talking about not allowing sheep to be transported or loaded if the ambient temperature is below zero degrees centigrade and yet no one’s saying: ‘Well, these sheep are out on the side of a hillside with … wind going, and the windchill factor is taking that down to minus 15 and they … adapted to be happy in those conditions. And is it maybe even worse to take an animal from those ambient conditions and put them in a lorry that has been warmed up to 5–10 degrees or whatever because we think* [it] *is comfortable?* [Roslag, industry].

In a similar vein, Serrai (government) observed, in reference to the EU’s vehicle air conditioning requirements: “*They think* [of] *the animal*[s] *as* [if] *the animal*[s] *were men.* [An] *animal doesn’t like to have cold … air*”.

Implicitly thinking of sheep as human-like could hinder our efforts to solve issues potentially linked to fatigue, such as acceptable transport temperature, hunger, and thirst. As to nipple drinkers, relevant in the context of the interplay between thirst and fatigue, participants dismissed these as not practical given their novelty and limited number. Cikta (industry) noted: “*[N]ipple drinkers, I mean, honestly: they’re not likely to know how to drink from anything like that*”. Examples of other participants’ comments include:

*It’s not realistic thinking that sheep can drink in a vehicle,* [that] *they go to a nipple and they use the nipple to drink. … First, they … don’t know what to do. Second, in a normal transport, on 4 decks … you can transport not less than 600 lambs, but how can you think that 600 lambs go to drink and they drink? I tell you, 10 second[s, t]hen they say: “please,* [it] *is your turn, now, please,* [it] *is your turn[,] now you”* [Serrai, government].

*[I]t’s a fairly common belief in the industry that sheep, especially if they’ve come off hill situations where the only water might be in streams or in big troughs, … probably won’t be used to drinking from little bowls or from nipples in vehicles. So, even if the water is provided, they may not recognise it* [Waziri, government].

No study documents sheep successfully using nipple drinkers on a moving truck (see the EFSA Opinion, in Nielsen *et al.* [2022]). Regulations were trying to address a real concern about thirst, but the result may be a requirement that is burdensome to comply with and does not solve the AW concern because it is not sufficiently tailored to sheep. As to air temperature control, EFSA Opinion Nielsen *et al.* (2022) notes the dearth of studies on the effects of ventilation within sheep transport vehicles, the effect of density on air’s ability to effectively regulate sheep temperature (as air needs space to move between animal bodies), and differences in temperature throughout the vehicle. Mandating measures that make sense in a human context without in-depth reflection from the animals’ point of view about what they mean in practice may not bring about the welfare outcomes the regulations sought to achieve (or may not be the most effective way of doing so).

#### “Not every stress is the end of the world”

This subtheme focuses on the view of a few participants that, with sheep, as with humans, some level of stress is not only not harmful, but can be adaptive and beneficial, and that life without stress is not natural. It was classified within the anthropocentric anthropomorphism theme because of the readiness with which some participants extended the belief that their lives need a certain degree of stress to feel full and real to the lives and experiences of sheep, without considering the nature of the transport experience as it may be perceived by a sheep:

*[I]f a sheep undergoes transport, for example, and initially they come off the vehicle and they look quite tired and a bit stressed and, not to relate it to a human experience because I think they’re very different, but you have a long day of work[,] but you can come home and relax and you* [are] *fine. I think there’s definitely a level of fatigue that sheep can cope with and that they’re built to cope with because that’s just everyday life* [Bibrik, AW scientist].

*As human beings, we aren’t free from stress. … if we look at the Five Freedoms, then from the last freedom around expressing natural state, stress is a part of that. If you haven’t experienced that, then you aren’t really living: you’re just in a gelatine state, where there’s no impact on your life ‘cause you don’t have to do any work and you got a tube down your throat that’s feeding you, etcetera, so there’s no external things, but that’s also not living* [Edilbay, industry].

*[A] little fatigue is maybe not … a problem … If I go on a holiday trip, I’m very tired …, but it’s OK, I can handle it. … Of course, these animals don’t go on a holiday, they will go to* [a] *slaughterhouse, but not every stress is the end of the world* [Gaddi, government].

While the paper is focused on fatigue, belief in the close connection between fatigue and stress is evident from some participants’ data. Jacob (AW scientist) notes: “*[A]ny stress factor* [will] *have an effect on the fatigue of the animals. So, every time that you are adding an extra factor to the animal, this has an effect on the general fatigue of the animal*”. Taleshi (industry) observes: “*[R]ecognise the stress … level within the sheep and do your best to reduce that, and if you reduce that, you won’t see fatigue in the sheep appear nearly as quickly*”. Another example:

*[T]he whole of the animal’s body is given over to trying to get over the stressors: its basal metabolic rate goes up; its heart rate goes up; heart rate variability goes down – the heart rate is constantly high. All of the things like that[,] … which will all tax the animal’s resources and lead to fatigue setting in earlier* [Polwarth, NGO].

There is no universally accepted definition of stress. Broom and Johnson (2019; p 4) define stress as “an environmental effect on an individual which overtaxes its control systems and results in adverse consequences and eventually reduced fitness”. Broom and Johnson (2019) reject the use of the term in relation to physiological changes such as hypothalamic-pituitary-adrenal axis activity and challenges resulting in positive outcomes (c.f. McEwen 2017), explaining that the former use is superfluous and unscientific, and the latter effectively equates stress with any stimulation.

Even assuming that the participants’ approach of viewing stress as potentially tolerable and adaptive is valid as to humans, transferring this perspective to the context of sheep transport is problematic. To some extent, humans have choice and control over activities that also cause stress (e.g. travelling, exercising) and may derive psychological satisfaction from having accomplished a stressful task (Broom & Johnson 2019). Sheep do not have a choice. Further, understanding a stressful event helps humans gain a sense of control (which is important to welfare) (Gamble & Creedy 2009). It is not possible to explain a stressful experience to a sheep, and not understanding what is happening may further burden sheep’s coping resources.

While Wickham *et al.* (2012) suggest that sheep can be habituated to transport, it is difficult to speak of transport’s adaptive value for the millions of animals going to slaughter which will not repeat the transport experience. It is not clear whether sheep can be habituated to on-farm stressors which they do encounter repeatedly, such as handling, and in particular shearing, sorting, and vaccination, as well as isolation, and the use of dogs (Hargreaves & Hutson 1990; Hutson & Grandin 2014). For example, Hargreaves and Hutson (1990) investigated whether repeated exposure to a stressor (sham-shearing) could lessen sheep stress response, finding only a slight reduction in the physiological and behavioural markers of stress following four instances of exposure to sham-shearing over a two-week interval (Hargreaves & Hutson 1990). Further, transport — combining multiple, potentially intense stressors — may be significantly more stressful than more mundane or isolated stressors.

#### They are “resilient until they’re not”

This subtheme suggests that the view expressed by most participants that sheep are resilient and stoic may amount to implicit anthropocentric anthropomorphism. We imagine how humans would react to a potentially negative experience such as fatigue and, not finding similar behaviour in sheep, prey species adept at hiding problems, we assume that there is no problem (Underwood 2002). An example of the commonly held belief that sheep are extremely hardy comes from Roslag (industry):

“*[S]heep have got an incredible ability to quickly recover from fatigue or stress or anxiety, and they can show signs of stress, and then as soon as they are put in a different situation, it’s like it never ever happened. … Just this time of year, you see sheep going through Caesarean section and as soon as … the lambs are out, they’re sewn back up, and they’re up, drinking and nuzzling the lamb, eating, like nothing ever happened.”*

Although resorting to anthropomorphism is natural where understanding is limited (Lockwood 1986), we cannot assume that there is no problem simply because we do not see it. Until relatively recently, in part due to the limited understanding of signs of pain, many animal species did not receive analgesia during surgery (Mogil *et al.* 2020). While efforts to better identify pain in sheep are underway (McLennan *et al.* 2016), analgesics continue to be underused for a host of reasons. By analogy, simply because we cannot yet reliably identify sheep fatigue (Cockram *et al.* 2012), particularly before it becomes exhaustion, we cannot assume that it is not present, particularly where sheep’s tendency to hide issues such as exhaustion is well-known (McLennan & Mahmoud 2019). In line with existing research, most participants acknowledged that sheep hide problems (including fatigue) until they reach critical levels. Alai (industry) observes: “*My view on sheep is they’re very, very stoic and don’t tell you that they’re suffering until quite late in their suffering. … [T]hey are very good at pretending they’re fine until the last minute*”. Other examples include:

*[T]hey’ll hide all of their symptoms as much as they can because there’s no benefit for them as prey animals to be displaying when they’re under stress, or panicked, or unwell. …[W]hen you’re actually clinically assessing a sheep, you will not know that that sheep is sick until it’s almost on death’s door …, because they just are really resilient until they’re not* [Bibrik, NGO].

*I certainly don’t think it’s easy to … see* [fatigue]*. …[T]hese are prey animals anyhow, so they’re gonna be disguising their behaviours as much as they can: if they’re ill, if they’re tired, … it’s inherent in them to disguise it until they’ve hit a point* [Lohi, government].

Knowing that sheep near exhaustion can seem fine but are just “*faking it*” (Drysdale, NGO), and being aware of the gaps in our understanding of how sheep express fatigue, means that we should give them the benefit of the doubt while we work to better understand their experience.

### See the whole animal

This theme advocates considering the whole animal when assessing potential AW problems such as fatigue. Existing guidance on identifying fatigue and exhaustion (Table 3) is brief and focuses more on what the animal is doing than on how it is doing it (Consortium of the Animal Transport Guides Project 2018). The focus on the individual behaviours more than on the animal performing it may be hindering our ability to identify fatigue.

**Table 3.** Current guidance on identifying fatigue/exhaustion

Behaviour	Expressive Quality
“Chin or limbs resting at partitions or troughs” “Closed eyes” “High drive to rest in recumbent position” “Inability/reluctance to rise” Consortium of the Animal Transport Guides Project (2018; pp 13, 45)	“General lethargy” “Apathy” “Lack of reaction” Consortium of the Animal Transport Guides Project (2018; p 45)

By contrast, when asked to describe a fatigued sheep or distinguish a fatigued animal from one that is not fatigued, most participants used colourful, evocative language going beyond the description of what the animal would be doing and highlighted how the animal would appear overall (Table 4).

**Table 4.** Examples of qualitative descriptive terms used by interviewees

Descriptors of Fresh Sheep	Descriptors of Fatigued Sheep
Alert Bright Bouncing Fighting alert Fresh Hop (v.) Jump (v.) Lively Look around (v.) Move quickly (v.) Relaxed Responsive Run (v.) Skip (v.)	Comatose looking Defeated (by exhaustion) Depressed Disorientated Dull Flop (v.) Hollow Listless Look like they’ve given up Look like they’ve done the journey Look of not being right Out of it Quiet Shuffle (v.) Slowing down demeanour Stagger (v.) Staring to the infinite (v.) Weak Zonked out Not with it at all
“(v.)” identifies descriptors formulated as verbs and verb constructions	

As humans, we constantly and intuitively make complex qualitative judgments in light of a broader context and our experience, taking account not only of what is done, but how it is done (Wemelsfelder 2007). And yet, historically, qualitative welfare assessment methods based on this innate human ability have been dismissed as unscientific (Wemelsfelder 2007). Qualitative methods such as QBA can and should be integrated in a systematic and thoughtful way, “deliberately and conscientiously applied through the use of formal methodologies” to help us better understand “what it is like to be that animal at a given moment in time” (Wemelsfelder 2007; p 20).

Qualitative methods have been used in a number of livestock species, including sheep, for individual and group assessments, and shown to be valid and reliable, with strong correlation to other AW measures (Wemelsfelder 2007; Wickham *et al.* 2012, 2015; Minero *et al.* 2016). Careful application of validated qualitative methods that consider “the whole animal” can also avoid erroneous anthropocentric interpretations (Wemelsfelder 2007; p 20).

Several participants noted that the same behaviour or lying posture could mean different things. For example, a sheep could be lying down in an “*alert*” or a “*zonked out*” way (Lohi, government). Further examples of evocative descriptions of fatigued and non-fatigued sheep are Najdi (NGO) describing fatigued sheep as “*panting, … [j]ust that general look of not being right, … dull eyes, listless looking-type, hanging head”*, as well as the following:

*[T]hey look like they’ve done the journey…. If I did a load of sheep from here, up the road … 4–5 hours, they’d all run down the tailboard and hop, skip, and jump, and out in the field … When you’ve done that trip for 12 hours, they just walk down the tail board* [Kooka, industry].

*[S]heep that weren’t particularly tired or stressed, … they’d be looking bright and alert and looking around them, [w]hereas if they’re totally exhausted … they probably stagger off the vehicle and then just flop down in any way[.] … Are they coming off bouncing down the tailgate and looking fairly bright or are they shuffling slowly down the tailgate and not being very responsive? …[I]f you saw them moving around …, you’d probably be able to realise that some sheep were fairly lively and moved quickly, who were fighting alert and looking around and responsive, and you might compare this with other sheep that were moving more slowly or weren’t responding so much* [Waziri, government].

Many participants’ focus on the qualitative aspects of sheep demeanour when describing fatigue suggests that it may be worth exploring the use of qualitative methods to examine “the expressive quality of animal behaviour and emotions” based on “an animal’s dynamic style of interaction with the environment” (Minero *et al* 2016; p 148). This improved understanding of fatigue could then be translated into better guidance for those tasked with assessing it in connection with transport.

The focus on physical health complicates identification of welfare problems in animals that do not show obvious issues; integrating qualitative assessment methods has been shown to address this point, resulting in a more comprehensive view of welfare (Wemelsfelder 2007). While prolonged observation, shown to facilitate interpretation of behaviour (Wemelsfelder 2007) may not be feasible in commercial transport, qualitative identification of fatigue on-farm or in lairage may yield useful information that could be applied in transport.

### Fatigue “never comes up”

This theme highlights that fatigue, not discussed to a meaningful extent with or by those charged with transporting sheep, may be an invisible problem. The concept of fatigue is present only peripherally in Regulation 1/2005. Although the EFSA Opinion in Nielsen *et al.* (2022) mentions fatigue, its definition is short and circular, and no robust scientific support is cited for most statements.

In regulations and, as noted by the majority of participants, in practice, the focus tends to be on other, more obvious issues, which may be perceived as more serious. Cikta (industry) notes: “[Fatigue is] *not a word that’s often bandied about*”. Alai (industry) confirms: “*[W]e don’t talk about fatigue in sheep. …We talk about if they look well or not, whether they’re well fed or not, and whether their skin looks like there are no parasites*”. Roslag (industry) states: “*[N]ever a topic coming from drivers … it very rarely comes up at processor level too, at the point of unloading, or at markets. And … I never hear that it’s a problem*”. Waziri (government) echoes: “*[W]e tend to think of animals with injuries, or animals that have exceeded the journey time in terms of being hungry or thirsty, but people tend not to think of fatigue as such*”.

Part of the problem, noted by several participants, may be identifying fatigue in commercial transport circumstances. Drysdale (NGO) notes: “*[T]ransporters, they’re just able to see things that are clearly visible, like extreme fatigue, pregnancy,* [or] *open wounds ….*”. Other examples include:

*[S]heep are probably one of the hardest to find a problem with, because …* [at] *four-five o’clock in the morning in any type of weather, good or bad, the driver’s under pressure to load those [400–500] sheep and get them gone* [Kooka, industry].

*I certainly have no easy way of picking out something that would be fatigued.* On identifying early signs of fatigue: *It would be a very difficult one[,]…and it would be … very difficult to quantify as, especially for a livestock driver who is seeing these sheep for the first time in either of their lives* [Taleshi, industry].

When asked whether fatigue is a real issue in commercial sheep transport, participant opinions ranged from the view expressed by a few NGO and AW scientist stakeholders that fatigue is a widespread, serious concern to one that it may be an issue for longer routes, to the view expressed by a few industry stakeholders that it is not a problem. Explaining their view that, in the context of long transport, all sheep are fatigued, Drysdale (NGO) observed: “*[W]e are talking about long journeys and knowing that the animals cannot … drink water … in such vehicles …, especially if this transport is going on during the summer, all of them are fatigued and exhausted, … if not for anything else,* [it] *is because of dehydration….”*

Most UK-based participants believe that fatigue is not an issue (at least within the UK). This might be related to the fact that most UK participants are industry stakeholders or to other factors, such as milder climate and stronger enforcement of transport regulation compliance. A few others noted that fatigue comes under consideration in instances where it may lead to a commercial loss. For example, Hampshire (AW scientist) noted: “*fatigue is considered a problem by industry for animals sent to slaughter, because it can mean that they will not be slaughtered, and therefore there will be what’s called lost revenue*”. Ultimately, however, for the most part, it is not discussed either because it is believed to be a non-issue or because other issues are perceived to be more urgent and important.

The overarching theme, “Human ‘spectacles’ are biased”, is about the fact that our view of other species is coloured by our individual background and our human-ness: “our human glasses are ingrained in us, and are very hard to remove (if possible at all). Nevertheless, if we are aware of having biased spectacles, we can attempt to address their effects upon us” (Rivas & Burghardt 2002; p 9). Although reflexive TA does not require that there be an overarching theme, here, one is appropriate. It is our human spectacles that colour our perception of how sheep experience transport and fatigue, including those aspects highlighted in the developed themes and subthemes. By rejecting anthropomorphism out of hand, we miss out on its potentially positive and productive uses. We also anthropomorphise without reflection, assuming animal needs to be the same as ours. Our human-centric view of the world leads us to consider behaviours of sheep piecemeal, without taking a broad view of the whole animal. Finally, it is our human view of the world that may prevent us from seeing fatigue in a species that may express it in ways very different to ours.

### Animal welfare implications

Fatigue can negatively affect AW and, left to progress to exhaustion, may render the animal non-recoverable. Our understanding of sheep fatigue remains limited: existing research and commercial transport conditions do not enable us to identify it before it becomes exhaustion. It is therefore likely that a potentially significant percentage of the millions of sheep transported within the EU annually suffer from fatigue, exacerbated by heat stress, overcrowding, and other conditions commonly present in commercial transport. The human-centric glasses with which we view the world can hamper our efforts both to understand and to address this problem, unless we are aware of the biases in our viewpoint and consciously work to overcome them or find ways to use them as practical jumping-off points to investigate issues relating to sheep fatigue.

This project’s analysis of stakeholder interview data has several AW implications. It illuminates the anthropomorphic nature of our thinking about sheep fatigue. Anthropomorphic thinking, in this case about sheep and their experience of fatigue (and, more broadly, live transport) underpins our behaviours, policy, and regulation. This can lead to positive or negative consequences. On the more constructive side, it can be used to persuade and inform, driving policy change (for example, around how and whether live transport continues). Drawing intelligent parallels between sheep and humans can also serve as a starting point for research to better understand sheep fatigue.

Anthropocentric anthropomorphism can cause us to miss potentially significant welfare problems or implement ineffective or damaging solutions to problems perceived by humans, which may or may not present a concern from a sheep’s perspective. This is important, as such solutions find their way into regulations and guidance, directly impacting sheep’s experience during transport. Questioning our thinking and focusing on understanding the animals’ *Umwelt* (“the environment as perceived by the animal” [de Waal 1999; p 265]) and arranging transport from those standpoints or significantly shortening or stopping it altogether can directly positively affect sheep welfare.

Interview analysis suggests further exploring qualitative methods (such as QBA) to better understand fatigue. The present approach (a short checklist focused on a few specific behaviours or postures associated with exhaustion) is too narrow. While the current toolkit for understanding sheep fatigue is limited, we may be able to expand it by harnessing innate human ability to observe and interpret complex circumstances to consider the broader context of how the animal is performing the behaviour. Finally, interview analysis also suggests that fatigue is not discussed in the context of live sheep transport. This is not surprising, given the limited body of science on the topic and its virtual absence from regulations. Acknowledging that a potentially important topic is absent from discourse is the necessary first step to remedying the problem.

In sum, the project contributes to the existing body of knowledge on issues relating to the perceptions around sheep fatigue during transport. It proposes new ways of advancing our understanding of this issue and engaging with stakeholders, which could help improve regulations and practice. These, in turn, may have a tangible positive effect on sheep welfare in transport.

## Conclusion

This paper sheds light upon a number of issues relevant to improving our understanding of sheep fatigue and its impact on AW in transport. It suggests conscious and thoughtful use of animalcentric anthropomorphism to drive research and influence policy. It cautions against insidious anthropocentric anthropomorphism, which may leave AW issues unaddressed or cause harm. It further advocates for a broader use of qualitative methods such as QBA to better understand fatigue. While these and other issues (e.g. sustainability; Baltussen *et al.* 2017) raised in relation to live transport are considered, a precautionary approach should be taken: we should assume that transport is fatiguing and, inter alia, seriously reconsider the current framework, including provided rest opportunities, the sufficiency of which has been called into question (Cockram *et al.* 1997; Cockram & Mitchell 1999; Nielsen *et al.* 2022).

## Supporting information

Colitti et al. supplementary material Colitti et al. supplementary material

## Supplementary material

The supplementary material for this article can be found at http://doi.org/10.1017/awf.2024.13.
