# Supplementary material for: Sheep fatigue during transport: Lost in translation?
Source: Anim Welf. 2024 Mar 11;33:e13. doi: 10.1017/awf.2024.13 (PMC10951664; doi:10.1017/awf.2024.13)
Supplement: Colitti et al. supplementary material [file S0962728624000137sup001.pdf]

Table S1. Final interview guide used in the study on understanding of fatigue in sheep during transport

A. **Background**

1. Current professional role
2. Familiarity with sheep

B. **Fatigue in sheep: understanding**

1. Fitness for transport: easy/difficult concept? (Note: This question was only put to the first few participants, as it quickly became evident that, given the time and space limitations of the project, fitness-related content would not be able to be included in the scope.)
2. Had to assess fatigue in sheep (or another small ruminant)?
3. Signs of fatigue in sheep
4. Is it easy or difficult to identify fatigue in sheep? Why?
5. How could we identify earlier signs?
6. Lying position – significance?
7. Lying location – significance?
8. Rumination/absence – significance?
9. Sentinel animals

C. **Implications for sheep welfare**

1. What impact could fatigue have on sheep welfare during transport?
2. Interplay between fatigue and other transport circumstances (e.g.,: journey time, stress, poor access to food or water, lack of resting, quality of transport/truck, handling, motion stress, novelty/sensory overstimulation, stop frequency).
3. Likely prevalence of undetected sheep fatigue
4. What could affect how quickly sheep recover when unloaded at a rest centre?

D. **Improvement**

1. Need to better understand fatigue in sheep?

2. If yes, how? If no, why?

E. **Closing**

1. Would you like to add anything else?
2. Anyone else I should speak with?

Table S2. The orientation of the reflexive TA used in the project in relation to main criteria of Braun and Clarke (2022)

Aspect	Explanation
**Theoretical Scheme** (realist/essentialist to relativist/constructionist)	A realist analysis seeks to identify the realities expressed in the data. A relativist analysis focuses on questioning and assessing them. This study used a critical realism approach. This approach is consistent with a view that there is an objective reality out there, but we cannot access it because our own perceptions and view of the world will always add a layer of subjectivity. In terms of epistemology (a theory pertaining to the concept of knowledge, "theorising what it is possible to know and meaningful ways of generating knowledge" (Bran and Clarke 2022, 166)), this study most closely aligns with contextualism, which "views knowledge, and the human beings who created it, as contextually situated, partial and perspectival" (Braun and Clarke 2022, 178).
**Participant Views** (experiential to critical)	An experiential approach focuses on the participants' own points of view. A critical approach seeks to analyse meaning as regards a specific topic or point. This study used a critical approach.
**Approach to Analysis** (inductive to deductive)	Inductive coding and theme development are driven by data content. Deductive coding and theme development are driven by the existing theoretical framework. This study used an inductive approach.
**Meaning Focus** (semantic to latent)	A semantic approach focuses on what is expressly said, while a latent approach – on what is implied. Neither is better than the other and both can be part of the same analysis. This study used a combination of both approaches.

Table S3. Phases of reflexive TA as applied to the interview data generated in the course of the project

Phase	Explanation
**Getting to Know Data**	Within a few days of each interview, KC watched the interview recording and simultaneously reviewed and corrected the transcript generated by the video recording software during the interview. The first three interviews were recorded using Zoom; the remainder were recorded using Microsoft Teams. Transcripts were downloaded from Zoom and Teams in Microsoft Word format and worked with further in that format. KC used her best judgment in deciding on appropriate punctuation. In the rare instances where interviewee speech was not discernible, KC inserted the following notation: "[inaudible]". This did not affect the analysis as none of the relevant segments contained inaudible language. KC read through the transcripts and noted some preliminary observations around common threads of meaning.<br><br>No member checking or participant validation (*ie* asking participants to check the accuracy of transcripts or the analysis and comment on whether they agree with it/it reflects their experience) was undertaken (Braun & Clarke 2022). These tools are designed primarily to mitigate the risk of interviewer bias in qualitative research (Ho & Limpaecher 2023). They are not universally-applicable to all types of qualitative research, and particular care should be taken using them in reflexive TA in which, as explained, interviewer/researcher bias is not avoided but rather acknowledged and integrated into the analysis (Braun & Clarke 2022). Their value is further reduced in projects such as this, in which analysis was undertaken not only at semantic but also at latent level, and a critical approach is used to analyse participant views (meaning that, among other things, participants may not recognise or disagree with the analysis, which does not undermine the validity of the analysis but does limit the utility or constructive value of participant validation) (see Table 2) (Braun & Clarke 2022).

Phase	Explanation
**Coding**	Coding refers to systematically going over each transcript to identify portions of text potentially relevant to the research question. There is no set length of text that should correspond to a code, not all text is coded, and some text could be coded more than once (if the same word grouping reflects more than one distinct idea). The main idea is that each chunk of coded text must reflect one idea, and that idea should be reflected in a shorthand description, a code. Moving through the text, KC created codes or re-used previously developed codes where appropriate. Coding was done fully electronically, annotating the transcripts using the Microsoft Word "comment" function.<br><br>KC started coding in early January, 2023, after the twelfth interview. KC coded the first twelve interviews in a continuous push across several days. The remaining six interviews were coded one-by-one, right after transcription. KC performed the second round of coding in the course of several days in mid-April, 2023. To keep track of any changes compared to the first round, KC used the "reply" function within each existing comment if the text had already been coded and added a new comment if the same text had not been previously coded.
**Generating Preliminary Themes**	In mid-April, when coding the transcripts for the second time, KC prepared a matrix with interviewee identifiers across the top and issues down the left side. KC identified issues as she went over the transcripts and reflected on codes potentially relevant to the research questions. With each successive interview coded, KC made notes in the relevant existing issue cells or added new issue cells. This matrix, once completed, facilitated the analysis both in terms of the prevalence of a particular idea within the data (though, as noted below, prevalence does not necessarily translate into interest or mean that a more prevalent idea is more worthy of becoming a theme) and in terms of thinking about grouping codes into broader themes relevant to the research questions.

Phase	Explanation
	Preliminary themes were discussed with MM and FL.
**Revising Themes**	KC started to revise themes in mid-April, 2023. KC repeatedly re-assessed, re-framed, and rearranged the initial candidate themes to ensure that they make sense within the framework of the entire dataset. The aim was to have each distinct theme "tell a convincing and compelling story about an important pattern of shared meaning" from the dataset and for the themes, taken together, to shed light on all important patterns relevant to each research question (Braun & Clarke 2022, p. 35).<br><br>KC, MM, and FL continued to discuss candidate themes and the thematic map.
**Further Refining Themes, Creating Thematic Map**	This stage focused on continued reflection on the analysis, theme refinement, tentative theme naming, and thinking about theme definitions/core ideas/boundaries (though all these were not finalised until after the report was completed).<br><br>KC prepared a first draft thematic map on April 20, 2023. Further versions followed on April 26 and 27; May 8, 26, 27, 28, and 31; June 1, 2, 13, and 14; and July 25. During this period, KC, MM, and FL exchanged e-mails and met via Teams to discuss the themes and the thematic map. The final thematic map includes 4 themes and 3 subthemes and shows how the themes and subthemes fit together to address the research questions. This number of themes and subthemes is consistent with Braun and Clarke (2022), which directionally notes that a thesis of similar scope would typically include 2-6 themes. Most theme names incorporate impactful phrases from participants.<br><br>While an overarching concept/theme is not required for quality reflexive TA, the analysis proposes one, namely the concept of biased human "spectacles" that colour our view of other species' behaviours, needs, *etc*., and whose effects we have to work to overcome (Rivas & Burghardt 2002).

Phase	Explanation
**Writing the Report**	While some writing occurred throughout the reflexive TA process (*i.e.*, starting with the notes on the first few interviews), more systematic report drafting started in early April, 2023. KC developed and refined the report continuously throughout the following months to ensure that it presents a thoughtful, in-depth examination of themes relevant to the research questions, together with "compelling, vivid data extracts" (Braun & Clarke 2022, p. 36). Data extracts were edited to (1) remove repetition of words (common in spoken conversation) and interjections and fillers (such as "umm", "so", "you know", "OK", "like", and similar); (2) correct grammar to improve readability and prevent identification (as many interviewees are non-native English speakers); and (3) shorten, taking care not to alter the meaning of the quoted extract (Braun & Clarke 2022).<br><br>To ensure confidentiality, each participant was assigned a code name corresponding to a breed of domestic sheep.<br><br>The number of quotes per participant ranges from one to five. While KC endeavoured to represent a range of stakeholder groups in discussing each theme, ultimately, the selection of quotes was subjective. It may have been influenced by the fact that some participants were not native English speakers and therefore may have found it more difficult to communicate their ideas with eloquence. Regardless of language fluency, some people are simply stronger communicators than others and may have expressed themselves in a more powerful way.
